# Embedding a safeguarding culture in research institutions: a multi-national partnership

**DOI:** 10.3310/nihropenres.14238.1

**Published:** 2026-04-24

**Authors:** Daniel Ansong, Mabuchi Banda, Sepo Mwikisa, Ijeoma Udeozo, Tara Tancred, Catherine Chunda Liyoka, Alex Osei-Akoto, Obiageli Nnodu, Elizabeth Mkutumula, Susie Crossman, Justin Pulford, Imelda Bates

**Affiliations:** 1 Kwame Nkrumah University of Science and Technology, Kumasi, Ashanti Region, Ghana; 2 National Health Research Authority-Zambia, Lusaka, Zambia; 3 University of Zambia University Teaching Hospital-Children's Hospital, Lusaka, Lusaka Province, Zambia; 4 University of Abuja, Abuja, Federal Capital Territory, Nigeria; 5 Centre for Capacity Research, Liverpool School of Tropical Medicine, Liverpool, England, UK; 6 Malawi-Liverpool-Wellcome Trust Clinical Research Programme, Blantyre, Southern Region, Malawi

**Keywords:** Safeguarding, International development, International research partnerships, Sickle cell disease, Sub-Saharan Africa

## Abstract

**Background:**

Research projects have a responsibility to safeguard their staff and research participants by treating them with respect and dignity and protecting them from harm. This is especially important in international development partnerships where substantial imbalances in power relations, resources and research governance structures are common. Demands for evidence and sharing of good practice about how international research partnerships can enhance safeguarding cultures in partners’ institutions are increasing. However, these partnerships are missing opportunities to amplify their projects’ impact beyond the project team and research focus.

**Methods:**

Our multi-national implementation research partnership is focused on patient-centred care for sickle cell disease in Africa and involves participatory approaches with community members and vulnerable patients. After establishing a project safeguarding plan and training, partners were keen to extend a safeguarding culture to their own institutions. We describe how our safeguarding trainers tailored the project’s approach and resources to introduce the concept of safeguarding in their institutions and set up training in four diverse contexts in Ghana, Nigeria and Zambia.

**Results:**

Lessons to apply to other research partnerships have been synthesised by comparing common factors which helped and hindered their efforts. These included jointly developing tools to identify safeguarding risks, using the projects’ establishing safeguarding processes, flexibility in the use of training resources, supporting institutionally influential safeguarding trainers, engaging institutional leaders as safeguarding advocates, embedding safeguarding within institutional systems, and planning for sustainability.

**Conclusion:**

Influencing institutional culture change to embed safeguarding approaches is a long process and is unlikely to be achieved within the lifetime of a short-term project. However, with careful planning and encouragement from research funders, the activities and resources within a research partnership can go well beyond simply delivering the primary research outputs. Even within 2–3 years they can be used to lever substantial impact on the safeguarding culture in partners’ institutions.

## Plain Language summary

Safeguarding people who participate in, and who carry out research, is especially important in projects involving international partners. Research participants and staff in poorer countries are especially at risk of being exploited due to differences in power relationships and the availability of resources compared to partners in more wealthy settings. Although safeguarding training and systems are now often included in multi-partner projects, opportunities are being missed to promote good safeguarding practice beyond the project across the partners’ institutions.

Our project on sickle cell disease involves partners in wealthy countries (US, UK) and sub-Saharan countries in Africa. Partners were keen to extend safeguarding practices across the African institutions and embed it into their pre-existing systems. We describe this process including factors that helped (e.g. committed institutional leaders) and the challenges encountered (e.g. lack of safeguarding policies). Although the short timeframe was insufficient to show long-term sustainability, the mechanisms the project staff used to help institutions take up and embed safeguarding practice illustrate how other projects could also achieve this through early planning and engagement of key influential actors.

## Introduction

### Safeguarding in a research context

Concerns around safeguarding in global health development have accelerated in the last few years following high-profile cases of abuse of vulnerable persons by international NGO staff [1]. Subsequently, guidance on good safeguarding practice in research has been introduced for those involved in funding and delivering research. The guidance emphasises the need to embed a safeguarding approach across institutions and projects. This means that research organisations are expected to develop and adopt a safeguarding policy that guides the processes to identify risks and prevent harm and have clear reporting mechanisms if harm occurs [2]. The aim is to promote a culture where safeguarding is an aspect of tailored, case-by-case planning for all individual projects. Organisations should therefore have:

• Institutional safeguarding policies, procedures, guidance and (confidential) records with ‘standing item’ oversight from a high-level board
• Designated and trained safeguarding officers to support staff and partners, and to monitor and advise on mitigating research-related safeguarding risks

Given the scarcity of published evidence on institutionalising safeguarding, there have also been calls for more sharing of experiences and learning about developing robust safeguarding policies and practices and how to embed these in research partnerships and in different contexts. ^1^ In this paper we share our experience of how our project’s safeguarding approaches and resources were levered to introduce a safeguarding culture across our research partners’ institutions.

### Safeguarding strategies in international research partnerships

Safeguarding challenges in research are shaped by power relations such as academic and professional hierarchies, and disparities in gender, age and socioeconomic status. These challenges are accentuated in research in global health and international development because of the large discrepancies in resources available to staff and students in higher-income countries compared to those in lower-income countries. In addition, formalised structures such as focal persons and reporting structures for safeguarding may not be well established in lower-income countries. This makes accountability mechanisms difficult and leaves little recourse for affected persons.

Safeguarding processes should aim to address power relations by building trust and centring the needs of the most vulnerable. International research partnerships - especially those that engage highly vulnerable persons - need to be particularly aware of safeguarding risks and abuses of power that may arise during research activities. To reduce the risk of harm and to promote positive and inclusive research environments, the partnerships should have safeguarding strategies and practices to ensure that everyone involved in the research is treated fairly with dignity and respect.

The UK Collaborative on Development Research (UKCDR) defines safeguarding as ‘taking all reasonable means to prevent harm from occurring; to protect people, especially vulnerable adults and children, from that harm; and to respond appropriately when harm does occur’. UKCDR states that ‘Everyone involved in the international development research chain, from research funders, planners and practitioners to local community members, has the right to be safe from harm’ [3]. ^2^ This includes all members of research teams (including support and administrative staff ) and communities and individuals involved in the research.

The need to protect the research team as well as research participants is often overlooked in safeguarding policies and practice. Researchers conducting fieldwork in the context of international research partnerships may face several challenges ( Table 1).

**Table 1.** Potential challenges faced by researchers.

Safeguarding challenges for research staff	Example
Physical safety	Crime, diseases, unsafe travel modes
Working conditions	Wage fairness, working hours, accountability
Emotional wellbeing	Fear, stress, isolation
Personal characteristics	Gender, sexual orientation, race
Role conflicts	Researcher’s ‘privileged position’, tension in data collection versus empathy and perceived duty of care
Feelings of guilt	Culture shock, disparity compared to participants’ wealth or living conditions
Sexual harassment	Sexual exploitation, abuse, discrimination
Power imbalances	Setting the research agenda, allocating resources, negotiating ownership of data and authorship
Political context	Travel bans, restrictions of visas/permits, data seizure
Ethics reviews	Multiple country applications, slow turnaround times

Within international research partnerships, understanding, recognition and approaches to safeguarding can vary widely among members. Some members may belong to institutions with clear and robust policies, whereas others may work in institutions with weak or no policies and with little exposure to safeguarding practice. Our PACTS [4] project involves research on patient-centred care for sickle cell disease and is a partnership among institutions in Ghana, Nigeria, Zambia, UK and US. Within the PACTS partnership, safeguarding policies and practices were especially limited in partners’ institutions in sub-Saharan Africa. The PACTS project was therefore viewed by these partners as an opportunity to influence and support the uptake of good safeguarding approaches in their own institutions.

Because of the significant differences in safeguarding understanding, capacity and experience among the PACTS partners, the project leadership not only invested time and resources in ensuring that there were robust safeguarding approaches within the project, but also established a network of safeguarding trainers across the partners and supported them in their ambition to promote the adoption of safeguarding policies and practices across their institutions.

Many funders now contractually require their awardees to have safeguarding guidance and processes in place and to monitor and report any incidents and awardees generally adhere to these requirements. However, we contend that many projects are missing opportunities to substantially extend their impact by promoting safeguarding approaches and training opportunities beyond the project itself to partner institutions.

To provide context we first describe how a safeguarding plan and approach was initially introduced across the PACTS project. However, the focus of our paper is on the novel approach we adopted to spread safeguarding awareness and training beyond the project. Our paper is an example of how the benefits of an international research partnership can be amplified to impact beyond the original project to influence the uptake of safeguarding by institutions and systems in partners’ countries.

## Methods

### Patient and public involvement

Patients and/or the public were not involved in the design, or conduct, or reporting, or dissemination plans of this research.

### PACTS project context

Our NIHR-funded project Patient-Centred Sickle Cell Disease Management in Sub-Saharan Africa (PACTS) (2022–2026) is a partnership among university teaching hospitals in Ghana, Nigeria and Zambia and academic institutions in the UK and the US. Central to our research are processes that facilitate collaboration between healthcare providers in our African partner countries and communities and individuals affected by sickle cell disease so that together they can identify problems and implement solutions to improve patients’ healthcare and wellbeing. Individuals living with sickle cell disease are viewed as especially vulnerable because they are often stigmatised and marginalised in their communities. As many community members were involved in our participatory approach as participants and co-researchers, developing a safeguarding plan was a start-up priority for our project team.

### Safeguarding planning within the PACTS project

To develop the safeguarding plan for the PACTS project we followed these steps adapted from Aktar *et al*. ^3^:

• Identify existing relevant national and institutional policies
• Ensure project teams understand the definition and principles of safeguarding
• Examine all project activities to jointly identify safeguarding risks and mitigations
• Develop and implement a safeguarding plan
• Jointly review and update the plan throughout the project lifetime

Existing policies in each country and/or the partners’ institutions that were specifically about safeguarding or that covered issues relating to aspects of safeguarding were documented.

*Training for project members on definition and principles of safeguarding*

A half day of face-to-face safeguarding training was provided for all sixteen-project staff who attended the PACTS start-up meeting using the Liverpool School of Tropical Medicine’s (LSTM) teaching resources adapted for the project context. LSTM was the project sponsor and therefore had overall responsibility for safeguarding. The training covered the purpose and principles of safeguarding. It emphasised that everyone has a role and responsibility for safeguarding and focused on discussions using real-life anonymised case studies from Africa-based projects.

*Identifying and reviewing safeguarding risks and mitigations*

At a subsequent workshop, project members were provided with a safeguarding risk template which included sections on, for example, documenting all vulnerable groups including staff, sub-contractors and community members; sensitive research topics; distribution of payments and resources; and safety of different work environments. All project activities were jointly reviewed and mapped onto the template and safeguarding risks for project staff and participants were identified. Mitigating actions for each risk (e.g. avoiding lone community visits and late-night working) and the person responsible for each risk were documented. The plan was implemented across the project and reviewed and revised quarterly by the PACTS management team.

### Facilitating roll out of safeguarding approaches to partner institutions

*Selection of safeguarding champions*

The PACTS lead researcher in each of the African partners’ countries identified an individual from their institution with suitable attributes to be their in-country project safeguarding champion. These attributes included being approachable and unbiased, having a commitment to sharing knowledge and advocating for safeguarding principles, and the ability to be supportive and work collaboratively. Zambia also selected an additional individual as their health research authority was keen to embed safeguarding at national level. The role of these individuals was to lead the implementation of safeguarding practices within the PACTS project. Although there was no requirement for partners to implement safeguarding training beyond the PACTS project, members were aware that PACTS provided an opportunity to strengthen safeguarding policies and processes across their own institutions. They therefore chose their safeguarding champions strategically to be individuals who were in influential positions in their institutions so they could advocate for the uptake of safeguarding approaches beyond the PACTS project.

*Upskilling safeguarding champions as safeguarding trainers*

The four safeguarding champions (one each from Nigeria and Ghana, and two from Zambia) initially received support from the PACTS UK team through monthly online discussions and a WhatsApp group was set up so they could share their safeguarding experiences. They underwent intensive training to equip them as safeguarding trainers during a one-week study attachment to a full-time safeguarding officer at the Malawi Liverpool Wellcome (MLW) Research Programme in Blantyre, Malawi [5]. The MLW centre is a regional leader in safeguarding, and the safeguarding team run training sessions across several African countries.

During their attachment at MLW, the PACTS safeguarding champions learnt how to provide safeguarding training for new staff and refresher training for existing staff. They attended a safeguarding committee meeting and met with safeguarding champions and the institution’s governance team to understand how safeguarding was embedded in institutional systems. They also visited research sites to see how safeguarding processes played out across different contexts and at levels of the research system. Through the safeguarding officer in Malawi, they were linked to a large virtual WhatsApp network of safeguarding trainers across several other African countries. These activities used a small proportion of PACTS project budget lines.

*Safeguarding trainers’ approaches for extending safeguarding across their own institutions*

During years 2 and 3 of the PACTS project, each safeguarding trainer (with support from their PACTS in-country lead researcher) developed and implemented their own strategies for rolling out safeguarding to their institutions. All of LSTM’s safeguarding resources [6] were made freely available for them to use however they chose, and they were also signposted to information about free additional online courses and materials. Each safeguarding trainer documented their experiences of introducing safeguarding concepts and approaches in their own institutions, and the successes and challenges they encountered. These were captured through the trainers’ own narratives, and in minutes of the regular in-country meetings of the PACTS team members and the cross-country management team meetings. The trainers’ experiences were compared, and lessons were synthesised about how to use an international research partnership to positively impact on uptake of safeguarding culture by partners’ institutions.

## Results

Information provided by the research trainers about how they advocated for and rolled out safeguarding beyond the PACTS project is summarised in four case studies. These describe the trainers’ activities, achievements and factors which facilitated or hindered the process.

### Kwame Nkrumah University of Science and Technology (KNUST) Kumasi, Ghana

The university introduced a safeguarding policy in 2018 to improve research culture and ensure best practices for safeguarding students, faculty and research participants. The safeguarding policy had been collaboratively developed by the Office of Grants and Research, the Quality Assurance and Policy Office, and the KNUST Counselling Centre who are jointly responsible for oversight. The policy recommends training for faculty and students and promotes a network of safeguarding trainers. However, by the start of PACTS in 2022 the policy had not been implemented as stakeholders to champion the policy’s operationalization had not been identified and there was a lack of safeguarding trainers.

The PACTS safeguarding trainer is a former Dean of the University’s School of Medical Sciences. After receiving safeguarding training through PACTS, he obtained endorsements from senior managers and identified individuals in different units across the university to act as safeguarding champions. This enabled him to tailor the safeguarding training to the needs of the stakeholders’ units as a starting point. Within six months of completing his own training, he had adapted the training materials and arranged training for 60 other safeguarding champions. These safeguarding trainers met with key stakeholders to develop a plan to implement the pre-existing safeguarding policy and to cascade safeguarding training across the university’s 85,000+ students and 5,000 staff. University leadership recommended that their existing counselling programme should be extended to encompass safeguarding training and provision, and responsibility for safeguarding has become incorporated into the remit of the university’s newly inaugurated Gender, Inclusion and Vulnerability Office. This office oversees embedding of ‘gender, inclusion and vulnerability responsiveness into teaching, research, leadership and everyday institutional culture’ [7].

Important challenges were the selection of appropriate safeguarding champions, scheduling and funding of training sessions, and making adaptations to institutional systems to embed safeguarding (e.g. counsellors’ job role adjustments; identification and training of departmental safeguarding champions). The process was facilitated by the existence of a safeguarding policy, the positive mindset and support of stakeholders, pre-existing awareness of some aspects of safeguarding, and institutional offices able to take responsibility for safeguarding issues. Planned next steps include securing ongoing funding, rolling out training across university departments, and consolidation of the administrative structures for implementation of safeguarding processes and incident reporting.

### National Health Research Authority (NHRA), Zambia

At NHRA, the safeguarding trainer is independent of the PACTS project and has a national position with responsibility for setting out the regulations and guidelines that govern the conduct of health research across Zambia within the Health Research Act (2013). The purpose of embedding safeguarding in national regulatory systems was to improve the research culture across the country’s health research institutions. The trainer delivered six sessions to 125 people on Good Clinical Practice which included sessions on safeguarding and how it linked with ethical research and confidentiality, respect, and accountability. Training also covered reporting pathways and the role of the safeguarding focal person to ensure timely and appropriate responses. Participants included individuals from ethics committees and from various research institutions around Zambia. Safeguarding is now embedded in the NHRA’s research capacity building curriculum. Next steps include ensuring safeguarding is a standing item on staff meeting agendas and research briefings, setting up a mentorship scheme for those handling safeguarding cases, evaluating safeguarding practices in ongoing studies, and periodic audits and training.

Roll out of the safeguarding programme has been facilitated by excellent support from the NHRA’s leadership team. The next challenges will be to secure funding to engage stakeholders beyond the health sector and to ensure that national policies and guidelines are extended to encompass safeguarding.

### University Teaching Hospitals - Children’s Hospital (UTCH), Lusaka, Zambia

The safeguarding champion at UTCH is a Public Relations Officer responsible for internal and external communications and is independent of the PACTS project. In her role she interacts with medical and non-clinical staff across the institution to improve awareness of safeguarding issues. This was the first safeguarding training in the institution and focused on creating a network of safeguarding champions across UTCH, with support from the senior leaders.

Training was initially provided for individuals from various departments across the hospital who had been selected as safeguarding champions. It emphasised the role staff play in creating a safe work environment and in helping to foster responsibility and accountability. Training was then extended to include community health workers, and the trainers also led a session on safeguarding at the national meeting of the Zambian paediatric association. In collaboration with NHRA, training materials were adapted to cover the intersection of healthcare delivery and research activities. The safeguarding trainer is now overseeing ongoing cascading training throughout the hospital. The hospital has policies that touch on some aspects of safeguarding such as bullying and harassment, and child protection, and the trainer is now advocating for a distinct institutional policy on safeguarding.

### National Centre of Excellence for Sickle Cell Disease Research and Training (CESRTA), Yakubu Gowon University (formerly University of Abuja), Nigeria

CESRTA conduct health research and provide generic training in topics related to the responsible conduct of research. The PACTS Nigerian safeguarding trainer has been appointed as CESRTA’s first safeguarding lead with responsibility for ensuring that safeguarding principles and best practices are integrated beyond the PACTS project into broader institutional policies concerning research ethics and culture. During the first year, the trainer conducted an audit of CESRTA’s existing policies and used the PACTS safeguarding risk matrix to identify policy gaps in relation to safeguarding. Although there was no specific safeguarding policy, relevant policies covered, for example, sexual exploitation, whistleblowing, and bullying and harassment.

The safeguarding trainer and the in-country research lead met senior management and other key stakeholders to secure high-level buy-in and endorsement of the safeguarding mandate. They also trained all staff involved in CESRTA’s projects and their onsite partners and enabled them to obtain formal certification in safeguarding. Following these foundational activities, the trainer’s focus shifted to integration of safeguarding mandates beyond CESRTA across the wider university’s policies. Navigating the complex governance structures and securing the necessary human and financial resources required meticulous effort and time. By adopting ethical safeguarding practices and embedding them into CESRTA’s grant applications and internal research operations, CESRTA is able to demonstrate its commitment to research excellence and integrity and the centre plans to be a role model for ethical conduct of research in the region.

### Comparison of experiences of rolling out safeguarding approaches to partner institutions

For all partner institutions, this was their first attempt at introducing the practice of safeguarding and implementing safeguarding training in their institutions. The position and seniority of the safeguarding trainers, and hence their diverse spheres of influence, was an asset. They had been purposefully selected to be well connected across their institutions and were able to influence senior management through their own institutional positions and those of their PACTS in-country research lead. A first step for all the safeguarding trainers was to engage key decision-makers in their institutions to get their endorsement and advice. They all reported that these stakeholders were very supportive of introducing safeguarding within their institutions because they wanted to improve their institutions’ research culture and integrity, and to adhere to good international practices.

The safeguarding trainers utilised the online safeguarding resources, case studies and other materials from LSTM and MLW and adapted these to suit their own needs. Examples of such adaptations included customisation for different cadres (e.g. human resources staff, clinical or laboratory staff, administrators and security staff ) to reflect national legislation and institutional policies. They drew on peer support from other safeguarding trainers across Africa through their WhatsApp groups. These were used to discuss workshops, policies and training materials, to share pre- and post-training assessments and to seek advice on, for example, low risk safeguarding concerns and appropriate reporting channels. In collaboration with colleagues from their institutions, they each provided safeguarding training tailored specifically for their institutions.

Although only one institution (KNUST) had a safeguarding policy, other partner institutions had some relevant policies. In addition to the general lack of specific safeguarding policies, other difficulties they encountered were in embedding safeguarding approaches within existing institutional systems and securing funding to sustain these changes and provide ongoing training in the long term. At the national level, the trainer in Zambia was able to incorporate safeguarding into the national regulatory systems but in other institutions, the institutional ‘home’ for managing safeguarding required negotiations and varied from research administration offices, a Quality Assurance and Policy Office to a Counselling Centre. Activities to address these challenges and to expand and consolidate the safeguarding training and systems across their institutions are planned for the final year of PACTS and beyond.

## Discussion

### Understanding safeguarding and laying the groundwork

The term ‘safeguarding’ is largely unrecognised outside high-oncome countries. ^4^ Although research staff from PACTS partners’ institutions in Africa were familiar with the need to keep participants safe, they were generally unaware of the broader remit of safeguarding to also protect the research team. This reflects a large review of ethics guidelines and protocols relating to research activities in low-and middle-income countries which revealed a general lack of focus on safeguarding in relation to research staff. ^1^

The PACTS project involved patients and their families in the research process, so it was particularly important to ensure that a culture of safeguarding was embedded throughout the research partnership. Although the PACTS leadership had a responsibility to abide by LSTM’s (the sponsoring institution) safeguarding policies, they did not impose a top-down approach to safeguarding. Instead, these issues were openly acknowledged and managed as a two-way learning opportunity. ^1^ This light touch and non-prescriptive approach enabled partners to navigate potential tensions between being able to hold all organisations to account regarding safeguarding without jeopardising the trusting and respectful relationship among partners.

Early in the PACTS project, time was invested in providing training, support and resources so that everyone was aware of their safeguarding responsibilities and how to report any issues. Project members were involved in co-developing the project’s safeguarding processes: this is recognised as important for building trust and genuine ownership and engagement. ^3^ Partners used the PACTS approach to safeguarding training and its practical tools (e.g. the risk matrix), and the way they were jointly developed and reviewed, as a ‘role model’ process that they could adapt for their own institutions.

### Common approaches to rolling out safeguarding: enabling factors and challenges

This was the first time that any of the project members from partner institutions in Africa had participated in training on safeguarding. As they became aware of how safeguarding policies and practices could enhance institutions’ research culture and environment, each African PACTS team decided to introduce the concept of safeguarding to their own institutions. They chose their safeguarding trainers strategically to be individuals with access to institutional systems and decision-makers and with support from their in-country PACTS research lead, each trainer was able to make progress in adapting and rolling out safeguarding training in their own institutions.

There were some common factors across the research partnership that facilitated this process. As a first step, the trainers all made strenuous efforts to engage high-level officials and get their endorsement. They used the PACTS platform as a role model and springboard for introducing safeguarding in their institutions and planned to start this within the lifetime of the project. The study visit to MLW in Malawi helped the trainers to consolidate their knowledge and skills, and it provided them with a social media network for peer support. They were able to adapt the PACTS’ safeguarding training materials and case studies to their own institutions’ needs and to identify mechanisms for sustainably embedding safeguarding in existing systems.

The strategies adopted by the safeguarding trainers resonate with the four building blocks that underpin organisational change ^5^:

1. ‘fostering understanding and conviction’ by engaging with institutional leaders so they could advocate for the introduction of safeguarding
2. ‘developing talent and skills’ by providing safeguarding training and setting up systems for cascading training
3. ‘role modelling’ the PACTS processes and working with institutional safeguarding champions to exemplify good practice
4. ‘reinforcing with formal mechanisms’ by identifying and addressing policy gaps and promoting safeguarding reporting systems in institutions

The safeguarding trainers also encountered several similar challenges. Specific safeguarding policies and strategies were lacking in most institutions, and they had to facilitate discussions about a suitable institutional office to be the ‘custodian’ of safeguarding. Both these factors potentially slowed down expansion of safeguarding across faculties and departments. All the trainers were concerned that in the longer term there may be insufficient institutional funds to support and sustain widespread cascading of safeguarding training. Despite these difficulties, all trainers succeeded in embedding a safeguarding culture and training in institutional centres or departments within two years of their own initial training.

### Safeguarding practice in international development research: lessons and principles

Lessons derived from the PACTS experience could be used by other international development research partnerships to enhance their impact beyond the initial project. These lessons ( Box 1) cover approaches a) within the project partnership for providing foundational knowledge and confidence in safeguarding, and b) that are relevant for expanding safeguarding beyond the project to provide additional impact across partners’ institutions.

Box 1. **Lessons learnt.**

*a) Provide safeguarding training and role-model processes within a research partnership*

**Establish good safeguarding practice across the project partnership from the start**

LSTM’s high standards for safeguarding training were reflected in the PACTS project which acted as a safeguarding ‘role model’ for Africa-based partners who were initially unfamiliar with the concept of safeguarding.

**Use real-life safeguarding scenarios as the focus of training**

Provision of basic safeguarding training for project staff and in particular, the use of real-life, relevant scenarios meant everyone could engage and learn equitably.

**Provide practical tools for managing safeguarding risks**

Joint discussions of safeguarding risks associated with project activities across all research sites using a standard risk matrix and how to mitigate these, highlighted the need for actions that might otherwise have been overlooked.

**Train safeguarding trainers as to lead safeguarding roll-out in each institution and facilitate their access to WhatsApp peer support groups**

Providing advanced training to develop a cohort of safeguarding trainers and networking them with other safeguarding trainers gave them confidence to lead cascading of safeguarding training in their institutions.

*b) Facilitating roll out of safeguarding approaches to partners’ institutions*

**Understand and build on the needs of institutions’ leaders to enhance their safeguarding approaches**

Each of the partners’ institutions had their own reasons for enhancing safeguarding practice which were generally based on a desire to improve institutional research culture and to comply with international good practice and funders’ requirements.

**Advocate for the development of institutional safeguarding-specific policies and processes**

Although institutions had some policies related to safeguarding, only a few had policies specifically for safeguarding. Advocating for development of institutional (and ideally national) safeguarding policies are important for institutional buy-in and to overcome hesitancy in implementing safeguarding approaches.

**Encourage adaptation and unrestricted use of all safeguarding training materials**

Allowing completely unrestricted use of training resources and encouraging adaption across contexts gave safeguarding trainers the ability to tailor safeguarding training for their institutions’ needs and ownership of their training programme.

**Strategically select institutional ‘influencers’ as safeguarding trainers**

Encouraging partners to be flexible in their choice of safeguarding trainers meant that they could strategically select individuals (including from outside the PACTS project) who were influential and well-connected in their institutions, and who also had appropriate characteristics to lead safeguarding roll-out.

**Engage with and involve decision makers in advocating for institutionalisation of safeguarding**

In-country teams prioritised engaging with institutional decision makers and co-developed plans with them for rolling out safeguarding independent of the PACTS project.

**Identify an institutional office to be the custodian of safeguarding in a research context**

Institutional leaders need to identify an office to take responsibility for oversight of safeguarding processes and training. This office should have a pan-institutional reach and be perceived as non-partisan and appropriate to lead safeguarding efforts. Examples from the PACTS safeguarding trainers included the National Health Research Authority, and offices/centres involved in counselling, research grants management, research training, quality assurance and public relations.

**Plan for sustaining gains in safeguarding made during a project’s lifetime**

Sustainability depends on securing funding for ongoing training and embedding safeguarding in systems across the whole institution. This is why the safeguarding trainers prioritised getting buy-in from decision-makers and identifying a safeguarding ‘home’ within their institutions. The lack of post-project to track if and how sustainability is achieved limits our ability to learn how to replicate durability in positive fostered initially through multi-partner projects.

There is no “one size fits all” in institutionalising safeguarding of research staff and participants, so it is not appropriate to develop specific protocols. ^4^ The approach used by the PACTS project to improve safeguarding provision aligned with several of the UK’s principles regarding safeguarding practice in international development research including ^1^:

• Safeguarding should be joined up within and between collaborating organisations
• Safeguarding should integrate and build on existing measures in UK research organisations and collaborating organisations
• Safeguarding should be approached in a spirit of inclusiveness and mutual learning
• The safeguarding process should be supportive, cognisant of power differentials and responsive to emergent needs

It has been suggested that in large institutions a two-level approach could be adopted for cascading safeguarding training with face-to-face training for leaders, managers and human resources staff and a mandatory online learning module for others. ^1^ Extending safeguarding training to non-staff members who also have research-related responsibilities, such as doctoral students, has also been advocated as part of embedding safeguarding in institutions. ^1^ This is reflected in our case studies in which training was extended to, for example, the national paediatric association and community health workers in Zambia and university counsellors in Ghana.

Interestingly, the NHRA in Zambia incorporated safeguarding into their national regulatory and ethical approval systems. There is some overlap between the concept and practice of safeguarding and research ethics frameworks (for example, regarding vulnerability and dependent relationships) and it has been suggested that the scope of ethics procedures could be expanded to include safeguarding [8]. Many higher education institutes already deal with safeguarding-related issues through their ethics committees and some have incorporated safeguarding as one of three fundamental pillars of research training for all, alongside data protection and ethics. ^1^

### Longer term impact evaluation

Safeguarding requires resources and time to build expertise ^1^ and it is ‘an iterative, ongoing learning journey’ ^3^: the PACTS safeguarding trainers are in the early stages of this journey. So far, they have focused on advocacy, engagement and training of safeguarding champions across their institutions. The next stages will involve developing or refining safeguarding policies, setting up safeguarding reporting mechanisms and evaluating the impact of these. They are aware that monitoring metrics should assess whether the process works for the individuals and institutions involved as well as quantitative data, such as the number of safeguarding reports once the systems is in ‘steady state’.

From the perspective of research funders and project managers, it is important to be able to track any changes in policies, knowledge and attitudes of research staff, and in the conduct of safeguarding processes in research. Learning how such changes can be achieved, and sharing these experiences, will enhance the impact and value for money of research partnerships. However, it takes 8–10 years for changes introduced through research projects to become sustainably embedded in institutional systems. ^6^ The short-term nature of project funding limits the ability to robustly track research infrastructure changes due to ‘the complexity of real-world research translation and evaluation’. ^7^

The importance of interventions to strengthen the capacity of institutions in all aspects of research governance and management, including safeguarding, is being increasingly recognised. ^8^ Project leaders need to be more aware that with foresight and early planning, they can substantially enhance a project’s impact on strengthening partners’ institutional systems that goes well beyond the primary research outputs. We have described how this can be done for safeguarding but similar approaches can be used for other aspects such as project management processes. ^9^ Researchers and research funders - with their traditional focus on the paradigm of training individuals in research skills - are missing opportunities to plan for and resource ^10^ these broader impacts on institutions’ research systems. ^11^ By planning strategically, sharing resources and expertise, and directing power towards institutions with weaker systems, it is possible for research partnerships to have much wider, lasting impact on reducing disparities and promoting research equity across institutions. ^12^

## Conclusion

The extensive roll out of safeguarding approaches across partner institutions was an additional positive outcome from our NIHR-funded project. Most research partnerships, especially those with a development focus, will have a safeguarding component and budget lines that can be used to support initial training and to co-develop safeguarding tools. With careful planning, a project’s activities and resources can be leveraged for much greater impact than just scientific research outputs. We have shown that extending safeguarding approaches to partners’ institutions is achievable within a project’s resources provided the safeguarding trainers are strongly supported by project partners and by their own institution’s leadership.

## Ethical approval

This study describes the process by which safeguarding training was established within an NIHR-funded project and how it was rolled out to institutions beyond the project. The process involved only project team members, and we did not collect primary data. Therefore, we did not need consent from those involved or ethical approval/waiver for this study.

## Data Availability

No data associated with this article.

## Footnotes

[1]
www.gov.uk/government/publications/charity-inquiry-follow-up-oxfam-gb-progress-on-safeguarding/charity-inquiry-follow-up-oxfam-gb-progress-on-safeguarding#conclusion

[2]
www.nihr.ac.uk/about-us/who-we-are/policies-and-guidelines/safeguarding-guidance

[3]
www.ukcdr.org.uk/safeguarding-prevention-from-harm/

[4]
www.pactsafrica.org

[5]
www.mlw.mw/news_and_events/mlw-staff-drilled-on-safeguarding/

[6]
www.lstmed.ac.uk/about/safeguarding

[7]
KNUST Opens Gender Inclusion and Vulnerability Office to strengthen policy implementation | Kwame Nkrumah University of Science and Technology

[8]
www.ukri.org/councils/esrc/guidance-for-applicants/research-ethics-guidance/research-with-potentially-vulnerable-people/
