# Supplementary material for: Introducing a safeguarding culture in research institutions: a multi-national partnership
Source: NIHR Open Res. 2026 Aug 6;6:30. Originally published 2026 Apr 24. [Version 2] doi: 10.3310/nihropenres.14238.2 (PMC13562927; doi:10.3310/nihropenres.14238.2)
Supplement: Peer review history

# Reviewer report: Reviewer response for version 2

**DOI:** 10.3310/nihropenres.15716.r41756

**Published:** 2026-09-28

**Authors:** Lennart Kaplan

**Affiliations:** 1 Georg-August-University of Göttingen, Göttingen, Germany

**Summary:**

 The PACTS project’s safeguarding strategy is implemented across multiple institutions in Sub-Saharan Africa, featuring a novel research team safeguarding component that strengthens ethical oversight. The article presents four case studies detailing implementation strategies, challenges, and best practices, emphasizing the importance of contextualized implementation. Long-term impacts and actionable learnings are discussed, highlighting as a potential for capacity transfer the areas of safeguarding and project management. The article is concise, well-structured, and uses sound language.

**Q1. Is the work clearly and accurately presented, and does it cite the current literature? (Partly)**

 I think the article is written clearly and in plain English. While I had the impression that it is well motivated, I felt that the reference to NGO's past neglect on safeguarding might be unclear for some readers and deserve 1-2 sentences explanation: Why do similar safeguarding needs matter for research? What are parallels of research (with NGOs, investigative journalism) and where are differences?

 Given that the safeguarding literature is constantly growing, I think the links to the nascent literature could be strengthened. I recommend checking Steinert et al. (2022) for a literature review, Abimbola (2019) for a pointed discussion of unequal research partnerships, and Schroeder et al. (2019) or the GRP's principles for hands-on guidelines and best practices.

 Finally, (but this is not a must and only a suggestion) I could see the implementation design getting much clearer by adding a figure or flow-chart on study design.

**Q2. Is the study design appropriate, and is the work technically sound? (Partly)**

 I liked the discussion section and have two suggestions how to further improve it. First, I missed a more critical discussion on any implementation challenges (e.g., was it always easy to get access to the high-profile champions or did it at times imply overburdening them?), reflexivity (how did internal power structures within PACTS shape implementation), and external validity (what do we learn about transferring safeguarding to other set-ups from the case studies). Second, I agree to another reviewer on measurable outcomes going beyond the number of people being trained. I think the authors do not need to come-up with other measures and would like to rather see a discussion on potential effectiveness measures in future and long-term studies.

**Q3. Are sufficient details of methods and analysis provided to allow replication by others? (Partly)**

 I think the method would gain in transparency by providing more detail on the trainings and the risk matrix in an appendix. In my opinion, this would also increase the contribution of the paper for other implementers.

**Q5. Are all the source data underlying the results available to ensure full reproducibility?**

 I support the suggestion of another reviewer (Bintu Mansaray), which provide clear guidance on how to approach data documentation.

**Q6. Are the conclusions drawn adequately supported by the results?**

 I felt the conclusions were generally supported, besides three points. First, I would like to see any reference or statistic on the statement "Many funders now contractually require their awardees to have safeguarding guidance and processes in place and to monitor and report any incidents and awardees generally adhere to these requirements." Second, the authors state that "Roll out of the safeguarding programme has been facilitated by excellent support" - I recommend to either tone-down or explain which factors made it "excellent". Third, the authors argue that the concept of "safeguarding" is not that known beyond the Global North. I think this statement should be qualified by discussing whether other concepts and terms may capture part of its role in the Global South (e.g., local laws, ethics). I do not recommend a full-fledged literature review. Instead I would rather like to see an acknowledgement that some mechanisms are already in place (though obviously not comprehensively).

 References:

 Abimbola, S. (2019). The foreign gaze: authorship in academic global health. *BMJ global health*, *4*(5), e002068.

 GRP (2025). 6 Principles of Research Ethics. Available via: https://globalresearch.scnat.ch/de/grp-guide/in_depth/research_ethics.

 Schroeder, D., Chatfield, K., Singh, M., Chennells, R., & Herissone-Kelly, P. (2019). Ethics dumping and the need for a global code of conduct. In *Equitable research partnerships: A global code of conduct to counter ethics dumping* (pp. 1-4). Cham: Springer International Publishing.

 Steinert, J. I., Atika Nyarige, D., Jacobi, M., Kuhnt, J., & Kaplan, L. (2021). A systematic review on ethical challenges of ‘field’research in low-income and middle-income countries: respect, justice and beneficence for research staff?. *BMJ global health*, *6*(7), e005380.

Is the work clearly and accurately presented and does it cite the current literature?

Partly

If applicable, is the statistical analysis and its interpretation appropriate?

Not applicable

Are all the source data underlying the results available to ensure full reproducibility?

No

Is the study design appropriate and is the work technically sound?

Partly

Are the conclusions drawn adequately supported by the results?

Partly

Are sufficient details of methods and analysis provided to allow replication by others?

Partly

Reviewer Expertise:

Global Health; Development Cooperation; Ethics and Equity of Primary Data Collection in the Global South

I confirm that I have read this submission and believe that I have an appropriate level of expertise to confirm that it is of an acceptable scientific standard, however I have significant reservations, as outlined above.

# Reviewer report: Reviewer response for version 2

**DOI:** 10.3310/nihropenres.15716.r41581

**Published:** 2026-09-09

**Authors:** Lorna Montgomery

**Affiliations:** 1 Queen’s University Belfast, Belfast, UK

I have reviewed this paper  in its current iteration and also paid attention to the previous reviewers’ comments  for the first version of the paper. I have  considered  how the previous reviewers recommendations have been addressed.

 To a large extent the revisions have strengthened the paper; revisions to this paper have ensured that it is more defensible, methodologically sound, and useful to international research teams facing similar setup challenges. I have made comments and suggestions for a small number of additional  edits in places

 Abstract:

 In the abstract, the background section could make a more explicit link between the importance of safeguarding  staff and research participants and amplifying their projects’ impact beyond the project team and research focus.

  Introduction:

  The introduction is largely clear. Shifting the context from general safeguarding in Low- and Middle-Income Countries (LMICs) to "research for development" is helpful and seeks to prevent overgeneralisation. This shift in emphasis  appropriately bounds the paper’s  findings to academic or research development partnerships rather than broader humanitarian or  community contexts.

 However, whilst definitions of safeguarding ,as outlined in the UK Collaborative on Development Research have been given, I think there needs to be additional information on  the concept of safeguarding in these contexts.  Some clearer identification of the risks within LMICs, with further exploration of risks to both children and adults would  provide necessary context and help ground the findings and discussion. This could be provided in an expanded ‘identifying and reviewing safeguarding risks and mitigation’ section.

  Methodology:

  The methodology section is clearer. Clarifying that the methodology relies on a "cross-case analysis" and detailing the processes of selection and characteristic of the trainers was helpful.

  However, explicitly addressing potential bias in self-reported trainer narratives and importantly, how these have been mitigated,  would be useful.

  Findings/ Discussion.

  Four very brief summaries of each case study have been provided, with useful follow on discussion.  Directly addressing the structural power hierarchies between high-income country funders and LMIC research partners helpfully strengthens the paper, identifying potential ethical issues. It was useful to include a lessons learned ( Box 1) section,  which will likely will enhance the paper’s impact.

 Acknowledging that the study captures only the initial activation phase, advocacy, infrastructure setup, and training rollout, should mitigate against  overreaching and protects the paper from valid criticism regarding unverified long-term outcomes

Is the work clearly and accurately presented and does it cite the current literature?

Yes

If applicable, is the statistical analysis and its interpretation appropriate?

Not applicable

Are all the source data underlying the results available to ensure full reproducibility?

No source data required

Is the study design appropriate and is the work technically sound?

Yes

Are the conclusions drawn adequately supported by the results?

Yes

Are sufficient details of methods and analysis provided to allow replication by others?

Yes

Reviewer Expertise:

NA

I confirm that I have read this submission and believe that I have an appropriate level of expertise to confirm that it is of an acceptable scientific standard, however I have significant reservations, as outlined above.

# Reviewer report: Reviewer response for version 2

**DOI:** 10.3310/nihropenres.15716.r41488

**Published:** 2026-08-31

**Authors:** Bintu Mansaray

**Affiliations:** 1 University of Oxford (Ringgold ID: 6396), Oxford, England, UK; 2 Institute of Gender and Children's Health Research, FREETOWN, Sierra Leone; 3 University of Bristol, Bristol, UK

I thank the authors for a thorough and constructive response. The revision engages seriously with the points raised, and several changes materially strengthen the paper,  in particular the scope statement anchored to NIHR's own safeguarding guidance, the recalibration of language around "embedding" and "culture change", the addition of limitations and reflexivity sections, the inclusion of training recipients' voices, and the deposition of the risk matrix, training materials and cross-site comparison as supplementary files.

 I accept two of the authors' respectful disagreements. First, the decision to scope the literature to safeguarding in health research for development, rather than safeguarding in low- and middle-income settings generally, is a legitimate scoping choice consistent with the paper's purpose. I offer one observation: the emerging critique of "safeguarding" as an Anglophone, donor-originated construct is not external to that scope, since the paper describes the transfer of a UK funder's safeguarding concept into four African institutions. A sentence engaging with this in the new reflexivity section would strengthen it, but I do not regard it as a condition. Second, I accept that outcome and impact evaluation was out of scope given the project timescale, and I welcome the signposting of future evaluation plans.

 I also welcome the clarification that MLW is an integral part of Kamuzu University of Health Sciences and a Malawian institution; this useful context was not clear in the original text, and I encourage its retention. I would gently note that it does not fully displace the observation about partnership architecture, since institutional integration into KUHeS and substantial Northern funding and affiliation are not mutually exclusive, but the new reflexivity statement is the appropriate place for that, and I am satisfied it is being addressed there.

  I consider everything else in my original report resolved. Congratulations to the authors.

Is the work clearly and accurately presented and does it cite the current literature?

Partly

If applicable, is the statistical analysis and its interpretation appropriate?

Not applicable

Are all the source data underlying the results available to ensure full reproducibility?

No

Is the study design appropriate and is the work technically sound?

Partly

Are the conclusions drawn adequately supported by the results?

Partly

Are sufficient details of methods and analysis provided to allow replication by others?

Partly

Reviewer Expertise:

Community paediatrician, public health specialist, Safeguarding lead in low-income settings; PSEAH and institutional safeguarding systems; North–South research partnerships.

I confirm that I have read this submission and believe that I have an appropriate level of expertise to confirm that it is of an acceptable scientific standard.

# Reviewer report: Reviewer response for version 1

**DOI:** 10.3310/nihropenres.15503.r40467

**Published:** 2026-05-21

**Authors:** Bintu Mansaray

**Affiliations:** 1 University of Oxford (Ringgold ID: 6396), Oxford, England, UK; 2 Institute of Gender and Children's Health Research, FREETOWN, Sierra Leone; 3 University of Bristol, Bristol, UK

Summary of the Article

 The paper reports on the PACTS (Patient-Centred Sickle Cell Disease Management in Sub-Saharan Africa, 2022–2026) project’s strategy for extending safeguarding beyond the project itself into the wider institutional systems of its African partners. After establishing a project-wide safeguarding plan (adapted from Aktar et al. 2020) and delivering a half-day training to project staff at start-up, the project identified one institutionally well-placed “safeguarding champion” in each African partner country (with an additional champion in Zambia for national-level work) and supported them through monthly online discussions, a WhatsApp peer-support network, and a one-week study attachment at the Malawi-Liverpool-Wellcome (MLW) Programme. These champions then designed and led their own safeguarding rollouts in their home institutions during years 2–3 of PACTS.

 The results are presented as four narrative case studies (KNUST, NHRA, UTCH, CESRTA) followed by a cross-cutting comparison. Across sites the authors identify enabling factors (institutional leadership endorsement; strategically chosen champions; flexible LSTM/MLW training resources; peer support via WhatsApp; pre-existing related policies; and willingness to embed safeguarding in existing offices such as counselling centres or research authorities) and persistent challenges (lack of specific safeguarding policies; identifying the institutional “home” for safeguarding; and funding sustainability). The discussion maps the strategy onto Basford and Schaninger’s four building blocks of organisational change and against UKCDR principles, and concludes that with deliberate planning, a project-level safeguarding investment can leverage wider institutional change within 2–3 years, even though full embedding is recognised to take 8–10 years.

**Q1. Is the work clearly and accurately presented, and does it cite the current literature? (Partly)**

 The paper is generally well written, and the case-study structure is easy to follow. However, several issues weaken the clarity and accuracy:

**The conceptual scope of “safeguarding” is unclear. **The UKCDR definition is cited up front, but the paper then moves between several distinct safeguarding domains, that is, PSEAH (protection from sexual exploitation, abuse and harassment), child safeguarding (particularly relevant given UTCH is a children’s hospital and many sickle cell patients are children), safeguarding of vulnerable adults, occupational safety/staff welfare, and research integrity, without clearly distinguishing them. Readers in low-income settings will note that “safeguarding” in NGO/humanitarian practice is dominated by PSEAH frameworks, while in clinical-research practice, it is closer to GCP and human-subjects protection. The paper would benefit from a short, explicit map of which domains it covers and which it does not.

**Engagement with safeguarding-in-LMIC scholarship is limited. **Aktar et al. (2020) and Kaplan et al. (2023) are cited, which is good. Relevant additional bodies of work that should be engaged include: Bond’s safeguarding guidance and self-assessment tools; CHS Alliance’s PSEAH framework; African Academy of Sciences / CARTA work on safeguarding and research ethics in African research institutions; the wider literature on PSEA in humanitarian and research settings (including community-based reporting mechanisms); and the emerging literature critiquing the term “safeguarding” as Anglophone and donor-driven. None of these needs lengthy treatment, but their absence narrows the paper’s framing.

**Calibration of language. **The paper uses “embedded”, “culture change”, and “safeguarding culture” interchangeably. These are strong claims. The evidence presented supports the more modest claim that safeguarding has been *introduced* and *institutionally located*, and that training has begun to cascade. “Embedded” should be reserved for situations where there is evidence of routinised use over time. Recommend tightening throughout.

**Plain language summary inaccuracy. **The PLS describes the partnership as involving “US, UK” countries and “sub-Saharan countries in Africa”, but the main text describes UK partner institutions (LSTM, MLW) without naming any US partner institution. Kindly include the US partner.

**Q2. Is the study design appropriate, and is the work technically sound? (Partly)**

 The choice to share practice-based learning is appropriate and welcome. The execution of that choice, however, lacks several elements that would normally be expected even for a reflective practice paper:

**No named methodological design. **The paper does not state whether it is a multiple case study, a process evaluation, an implementation research study using a recognised framework (CFIR, RE-AIM, EPIS, etc.), or a narrative inquiry. From a safeguarding specialist's standpoint, a multiple-case study with cross-case synthesis, or a most-similar systems comparative case design, would be the most natural fit and could be implemented retrospectively.

**Self-report and author/subject conflation. **The safeguarding trainers are also among the authors and are also the data subjects. The narratives that constitute the primary evidence base are therefore not independent. This is an important methodological limitation that should be acknowledged explicitly, with reflection on how it may have shaped what was captured (e.g., publication-friendly framing of successes, underreporting of frictions or failed attempts, omission of sensitive incidents). I would emphasise that, for a paper about safeguarding, the limitation matters doubly, because under-reporting and overly positive framing are well-documented risks in safeguarding self-assessments.

**No triangulation or independent voices. **The paper would be strengthened by even brief inclusion of perspectives from training recipients (How did they experience the training? Did they feel safer? Did they know how and to whom to report?), institutional leaders (Did they consider the work a priority and why?), and most importantly, community members or people living with sickle cell who were part of the participatory research process. A small number of anonymised quotes drawn from existing project records would help.

**Outcome measurement is thin. **The technical soundness of a safeguarding intervention should not be judged by training numbers alone but by signals of behavioural and systemic change: have any safeguarding concerns been raised through the new mechanisms? How were they handled? Were the people who raised them satisfied? The paper presents activity-level outputs (people trained, policies advocated for, materials adapted) but very little on outcome-level signals. If outcomes are deliberately out of scope for the current paper, please state this and signpost a future evaluation plan.

**The ethical approval claim should be revisited. **The authors state that no ethical approval was required because no primary data were collected. From a safeguarding perspective, this is questionable: trainers’ written narratives are **primary data; the case studies name identifiable individuals, e.g., “the former Dean of the School of Medical Sciences”, the “Public Relations Officer” at UTCH and identifiable institutions; and the case studies disclose institutional weaknesses (lack of policy, lack of funding, governance complexity) that could be sensitive. At a minimum, the paper should describe the consent and review process used (written consent from named individuals and institutional sign-off from each partner), and ideally should reflect on whether an institutional review or ethics waiver process should have been sought. 

**Reflexivity on power within the partnership. **The paper is at its best when it acknowledges the “light touch” approach that LSTM adopted, but the partnership architecture remains that of a UK-sponsored, UK-trained, MLW-mediated cascade. The South-South relay through MLW is itself an institution with strong Northern affiliations. A short, honest reflection on this would strengthen rather than weaken the paper, and is consistent with the safeguarding principle of recognising power.

**Q3. Are sufficient details of methods and analysis provided to allow replication by others? (Partly)**

 The process descriptions of how the project safeguarding plan was developed, how champions were selected, and how the MLW attachment was organised are sufficiently detailed to be useful. However, several elements that another team would need to replicate this work are missing:

**Selection criteria. **“Suitable attributes” for safeguarding champions are described in general terms, but not as operational criteria or as a documented selection process. A short box or appendix specifying the criteria and how they were applied would be valuable.

**Training content. **“A half day of face-to-face training” for project staff; “one-week attachment” at MLW; “six sessions to 125 people” at NHRA is highlighted, but content is not. Please include a simple summary of the session's learning objectives, the duration of each module, and the assessment approach (pre- and post-tests are alluded to but not described) for replication.

**Risk matrix and tools. **The “safeguarding risk template”  or risk matrix is central to the methodology and is referenced by trainers as the tool they used in their institutional work. It is, however, not provided. LSTM’s and MLW’s materials are referenced via URLs but not enumerated. As a minimum, a sample of the risk matrix and any adapted tools should be published as supplementary material.

**Data collection and analysis. **“Trainers’ own narratives” and “minutes of regular meetings” were the source data. There is no description of how these were collected (template? interview? free-form?), who synthesised them, or how lessons were derived (thematic analysis? framework analysis?). A short methods paragraph specifying these would address both this question and Q2.

**Q5. Are all the source data underlying the results available to ensure full reproducibility? (No)**

 The “Data availability” statement reads “No data associated with this article”, yet the paper references multiple data sources (trainers’ narratives, meeting minutes, pre/post training assessments, risk matrices, training materials, WhatsApp peer-support exchanges). I don't recommend releasing raw narratives or WhatsApp content, as some of it may be personally identifiable or pose safeguarding risks. However, the following should be deposited in an appropriate open repository (e.g.,  the NIHR Open Research data linkage, or the project’s institutional repository), with clear documentation of any redactions and a brief rationale for any items that have been withheld:

 a. An anonymised version of the safeguarding risk matrix template.

 b. A summary of the training curriculum, learning objectives and pre/post-assessment instruments.

 c. The data-collection template/prompts used to elicit trainers’ narratives.

 d. A simple cross-site comparison matrix of enabling factors and barriers.

 e. A statement of what cannot be shared and why (e.g., individual case notes; disclosures). The current blanket statement doesn't fully reflect what the team can reasonably make available and doesn't meet the openness expected by NIHR Open Research.

**Q6. Are the conclusions drawn adequately supported by the results? (Partly)**

**Supported. **The conclusion that with deliberate planning a project can begin to lever wider institutional safeguarding work within 2–3 years is reasonably supported by the case studies, which describe concrete activities (policy operationalisation at KNUST, national-level integration at NHRA, first-ever training at UTCH, audit and certification at CESRTA).

**Not yet supported. **Claims of “substantial impact on the safeguarding culture in partners’ institutions” overstate what the evidence shows. Culture change cannot be inferred from training delivery and policy advocacy alone. The paper itself acknowledges that 8-10 years are needed for sustainable embedding. Recommend re-wording to claim impact on the foundations of a safeguarding culture, while reserving “embedded” and “culture change” for future, outcome-based publications.

**Missing safeguarding outcomes question. **A safeguarding programme that has trained hundreds of people across four institutions and yet reports no signal whatsoever about disclosures or incidents handled is a finding in itself. Has anything been reported through the new mechanisms? If yes, how was it handled (in aggregate, anonymised terms)? If no, what does the team infer from that absence: a trust gap, a reporting-channel gap, or a genuine absence of incidents? Either answer is informative, and engaging with it would substantially strengthen the conclusions.

Is the work clearly and accurately presented and does it cite the current literature?

Partly

If applicable, is the statistical analysis and its interpretation appropriate?

Not applicable

Are all the source data underlying the results available to ensure full reproducibility?

No

Is the study design appropriate and is the work technically sound?

Partly

Are the conclusions drawn adequately supported by the results?

Partly

Are sufficient details of methods and analysis provided to allow replication by others?

Partly

Reviewer Expertise:

Community paediatrician, public health specialist, Safeguarding lead in low-income settings; PSEAH and institutional safeguarding systems; North–South research partnerships.

I confirm that I have read this submission and believe that I have an appropriate level of expertise to confirm that it is of an acceptable scientific standard, however I have significant reservations, as outlined above.

# Response

**Published:** 2026-07-29

**Authors:** Susie Crossman

We would like to thank you for the time you have taken to carefully review our paper. Your helpful comments and suggestions, which we have incorporated, have significantly improved the paper. Responses to your specific suggestions are below.

  

1. We have added a statement explaining the conceptual scope of ‘safeguarding’ that we have used in the paper. This is based on the safeguarding guidance from our project funders, the National Institute for Health Research ( Preventing Harm in Research: Safeguarding guidance | NIHR). In addition to the general principle of treating everyone with respect and dignity, our scope for safeguarding incorporates several distinct domains including sexual exploitation, abuse or harassment; child protection; and equity irrespective of protected characteristics. Where we mention, for example, sexual exploitation, abuse or harassment, or child protection, we have made it clear that these are within the conceptual scope of safeguarding.
2. Our study focuses on safeguarding in the context of health research for development rather than safeguarding in LMIC more generally (see response #1) and this is reflected in the references we have cited. However we have now expanded these to include some additional references suggested by the reviewer.
3. Our study describes the early stages of introducing the concept of safeguarding and establishing training, processes and policies. We agree that it is too early to provide evidence that these activities have been embedded securely in institutional systems or that there has been a substantial culture change. We have therefore adjusted the language throughout as suggested by the reviewer making it clear that a safeguarding culture has been embedded within the PACTS project partnership but not yet across the partners’ institutions. In summarising our findings we have explained that ‘within 2-3 years the [research] partnership can be used to introduce the concept of safeguarding and to establish training cascades across institutions’ and acknowledging that it will take longer than this for safeguarding processes to be fully operational.
4.  We have clarified that the role of the US-based PACTS project partner was to lead a ‘journalism workstream’. They were not directly involved in this safeguarding study. We have also clarified that MLW is an integral part of the Kamuzu University of Health Sciences, Blantyre and is a Malawian institution.
5. We appreciate the suggestion to describe the methodological basis for practice-based learning as a cross-case analysis and have now included this in the methods section of the paper.
6. We have added a specific section on limitations where we explain potential biases due to self-reporting and how we tried to mitigate these, including triangulating information against meeting minutes. We have now included a reflexivity statement on the partner power dynamics and how we addressed these hierarchies.
7. (also see #9 and #14). The safeguarding trainers have not yet formally evaluated the use of the safeguarding processes as they have not yet been established in the institutions. However they have collected feedback from those involved in the training and we have included quotes from some of these ‘independent voices’ within the paper.
8. We recognise that outcome and impact evaluations - such as whether individuals that use the safeguarding processes are satisfied, trends in reporting of safeguarding issues, policy development and changes in knowledge and attitudes of research staff - are important to measure in the longer-term. We have highlighted this in the section on impact evaluation but the timescale was too short to generate any meaningful data on safeguarding reporting practices so this was out of scope for the current study. However the safeguarding trainers and their institutions are developing evaluation plans to capture such metrics and we have updated this information in the text. The purpose of our study was specifically to describe the early stages of setting up safeguarding systems (i.e. advocacy, engagement, training of safeguarding champions and cascading training) to generate practice-based learning and to show that this can be done within a typical project time frame.
9. We understand that it is not possible to de-identify some of those involved in this study as the safeguarding implementation team are authors. The study team therefore agreed not to be anonymised and the information they provided was selected by them and used with their consent. We have made sure that everyone else involved is not identifiable. Ethics approval was given by the appropriate national and institutional boards for the overall PACTS project. We have reflected this in the revised ethical approval statement.
10. Reflexivity – see #12
11. The selection of safeguarding champions was an informal process and was done differently by each partner based on the personal and professional attributes described in the paper.
12. The content of the safeguarding training for trainers, pre- and post-assessments and an accompanying resource list are now included as supplementary files 3 and 4. The content of the trainers’ training is what they each adapted for subsequent cascading of training tailored for their own institutions, supplemented by material from the resources list.
13. The safeguarding risk matrix has been included as supplementary file 1
14. More detailed information has been provided about the trainers’ narratives (including the prompts used to elicit these) and data from the meeting minutes. A supplementary file (#2) is now included to show a cross-site comparison of enabling factors and barriers/challenges
15. We have shared the risk matrix, training programme, assessments and resources, and created a cross-site comparison matrix as supplementary files 1-4 as suggested by the reviewer. Other issues have been addressed within the paper.

# Reviewer report: Reviewer response for version 1

**DOI:** 10.3310/nihropenres.15503.r40463

**Published:** 2026-05-13

**Authors:** Lesong Conteh

**Affiliations:** 1 London School of Economics and Political Science, London, UK

Congratulations to the authors on writing a paper that addresses an under-explored and important topic. Below are suggested areas for strengthening content:

 Background

1. If I understand correctly, the introduction conflates safeguarding in research with safeguarding in international development more broadly. The opening references to high-profile NGO abuse cases provide useful context but the paper is specifically about research safeguarding. A clearer distinction between these two domains early on would sharpen the framing. Or maybe they are two sides of the same issue?

Methods

1. The methods section describes what the project did, but does not situate this specific paper within a recognised methodological framework. Is this implementation research, a case study analysis, or a process evaluation? The authors use the term "implementation research partnership" in the abstract but apply no implementation science framework to the analysis. Greater methodological clarity is needed.
2. The McKinsey four building blocks framework appears in the discussion but is not used to structure the analysis or case studies. Suggest the authors either apply it systematically throughout or replace it with a more appropriate analytical framework from the implementation science or organisational change literature.

Results

1. The paper promises a comparative synthesis but delivers largely parallel narratives. A structured comparison, for example a matrix of enabling factors and barriers across the four sites, would make the analysis more rigorous and considerably more useful to readers seeking to apply these lessons elsewhere.

Discussion & Conclusions

1. The safeguarding trainers documented their own experiences, and these accounts form the primary evidence base. The paper does not acknowledge self-reporting as a limitation or discuss how it may have shaped what was captured. This should be addressed in the limitations section.
2. It is outside my area of expertise, but the paper may benefit from considering whether there are relevant national or international legal frameworks through which safeguarding obligations could or should be interpreted. If this dimension exists, engaging with it could strengthen the policy relevance of the paper.
3. Were there any lessons that UK or US institutions took back from this experience? Reflecting on bi-directional learning this would strengthen the paper.

Is the work clearly and accurately presented and does it cite the current literature?

Yes

If applicable, is the statistical analysis and its interpretation appropriate?

Not applicable

Are all the source data underlying the results available to ensure full reproducibility?

Partly

Is the study design appropriate and is the work technically sound?

Partly

Are the conclusions drawn adequately supported by the results?

Yes

Are sufficient details of methods and analysis provided to allow replication by others?

Partly

Reviewer Expertise:

Health economics, diagnostics, and health systems

I confirm that I have read this submission and believe that I have an appropriate level of expertise to confirm that it is of an acceptable scientific standard.

# Response

**Published:** 2026-07-29

**Authors:** Susie Crossman

We would like to thank you for the time you have taken to carefully review our paper. Your helpful comments and suggestions, which we have incorporated, have significantly improved the paper. Responses to your specific suggestions are below.

  

1. We have clarified that the main focus of the paper is on safeguarding in the context of health research for development so we have drawn on references from both development and research literature.
2. We have added a statement to explain that it is the research project (PACTS) that uses implementation research (i.e. not this safeguarding study).
3. We have included a matrix comparing the enabling factors and barriers across the four sites as supplementary file 1.
4. We have included a section outlining the limitations of self-reported findings and how this may have influenced the data capture and interpretation
5. There are legal frameworks and policies (covering for example, child protection and various forms of discrimination as mentioned in the paper) in the partner countries. These include The Children’s Code Act, 2022 (Zambia), Child's Rights Act 2003 (Nigeria) and Children's Act 1998 (Ghana). Anti-discrimination protections are included in the constitutions of Ghana (1992), Nigeria (1999) and Zambia (2016 amendment).  However these frameworks only partially cover all the aspects and obligations concerning safeguarding.
6. The lessons and principles we have outlined for safeguarding practice in international development research summarise the learning for all those involved in research partnerships. This learning is multi-directional irrespective of whether partners are from the global ‘north’ or ‘south’ and we have explained this in the text.
